# Complete Genome Sequence of Serratia marcescens Podophage Pila

**DOI:** 10.1128/MRA.01420-19

**Published:** 2020-01-02

**Authors:** Loraine Melbern, Kathryn Broussard, Russell Moreland, Mei Liu, Jolene Ramsey, Justin Leavitt

**Affiliations:** a Center for Phage Technology, Texas A&M University, College Station, Texas, USA; DOE Joint Genome Institute

## Abstract

Multidrug-resistant Serratia marcescens strains cause serious nosocomial infections in humans. Here, we present the annotated genome sequence of S. marcescens podophage Pila. Similar to its closest relative, *Enterobacteria* phage T7, Pila has a 38,678-bp genome, predicted to encode 51 protein-coding genes, and contains 148-bp direct terminal repeats.

## ANNOUNCEMENT

Serratia marcescens, a facultative, Gram-negative bacterium of the family *Enterobacteriaceae*, causes nosocomial infections in severely immunocompromised or critically ill patients (1, 2). S. marcescens is commonly found in water, soil, animals, insects, and plants, and multidrug-resistant strains have emerged (3). Here, we present the isolation and genome annotation of S. marcescens phage Pila.

Bacteriophage Pila was propagated on host Serratia marcescens strain D1 (no. 8887172; Ward’s Science) aerobically at 30°C and 37°C in LB broth and agar (BD). This phage was plaque purified, via the soft-agar overlay method (4), from filtered (0.2-μm filter) municipal wastewater collected in College Station, Texas. Phage genomic DNA was prepared as described by Summer (5), using a modified Promega Wizard DNA clean-up system shotgun library preparation protocol. The phage DNA library was prepared with an Illumina TruSeq Nano low-throughput kit and then sequenced on an Illumina MiSeq system with paired-end 250-bp reads, using a 500-cycle v2 kit. FastQC (www.bioinformatics.babraham.ac.uk/projects/fastqc) was used for quality control of the 4,413 total reads in the phage-containing index, and reads were trimmed with the FastX Toolkit v0.0.14 (http://hannonlab.cshl.edu/fastx_toolkit). SPAdes v3.5.0 was used with default parameters to assemble a single contig at 14.9-fold coverage (6). The contig was confirmed to be accurate at the termini by Sanger sequencing of PCR products amplified from the contig ends (forward, 5′-AAGGCTGTAAGTGACGCTATTG-3′; reverse, 5′-TAGCCTCAGGTGAAGCTGTA-3′). Protein-coding genes were predicted using GLIMMER v3.0 and MetaGeneAnnotator v1.0 (7, 8). Potential tRNA genes were assessed with ARAGORN v2.36 (9). Rho-independent termination sites were annotated using TransTermHP v2.09 (10). Gene function was predicted using InterProScan v5.33-72, TMHMM v2.0, and BLAST v2.2.31 searching the NCBI nonredundant and UniProtKB Swiss-Prot and TrEMBL databases, with a maximum expectation value of 0.001 (11–14). Genome-wide DNA sequence similarity was calculated using progressiveMauve v2.4.0 (15). All analyses were conducted using the Center for Phage Technology (CPT) Galaxy and Web Apollo instances (https://cpt.tamu.edu/galaxy-pub), using default parameters unless otherwise stated (16, 17). Phage morphology was determined using negative staining with 2% (wt/vol) uranyl acetate and transmission electron microscopy performed at the Texas A&M University Microscopy and Imaging Center (18).

The 38,678-bp double-stranded DNA genome of podophage Pila has a G+C content of 48.8%. The 51 predicted protein-coding genes correspond to a coding density of 92.5%. Notably, Pila shares 83.7% nucleotide identity with *Enterobacteria* phage T7 (GenBank accession no. AY264774). PhageTerm analysis predicted the genomic termini to be 148-bp direct terminal repeats, typical for T7-like phages (19), which is where the Pila genome was reopened. More than 40 Pila proteins have significant similarity to those of two *Yersinia* phages, YpP-R and YpsP-G (GenBank accession no. JQ965701 and JQ965703, respectively).

### Data availability.

The genome sequence and associated data for phage Pila were deposited under GenBank accession no. MN098329, BioProject accession no. PRJNA222858, SRA accession no. SRR8892142, and BioSample accession no. SAMN11408657.
